# Ultrasensitive SERF atomic magnetometer with a miniaturized hybrid vapor cell

**DOI:** 10.1038/s41378-024-00758-6

**Published:** 2024-08-30

**Authors:** Yintao Ma, Yao Chen, Mingzhi Yu, Yanbin Wang, Shun Lu, Ju Guo, Guoxi Luo, Libo Zhao, Ping Yang, Qijing Lin, Zhuangde Jiang

**Affiliations:** 1 State Key Laboratory for Manufacturing Systems Engineering, International Joint Laboratory for Micro/Nano Manufacturing and Measurement Technologies, Xi’an Jiaotong University (Yantai) Research Institute for Intelligent Sensing Technology and Systems, Xi’an Jiaotong University, Xi’an, 710049 China; 2 School of Mechanical Engineering, Xi’an Jiaotong University, Xi’an, 710049 China; 3 School of Instrument Science and Technology, Xi’an Jiaotong University, Xi’an, 710049 China; 4 Shandong Laboratory of Yantai Advanced Materials and Green Manufacturing, Yantai, Yantai, 265503 China

**Keywords:** Electrical and electronic engineering, Optical physics

## Abstract

The chip-scale hybrid optical pumping spin-exchange relaxation-free (SERF) atomic magnetometer with a single-beam arrangement has prominent applications in biomagnetic measurements because of its outstanding features, including ultrahigh sensitivity, an enhanced signal-to-noise ratio, homogeneous spin polarization and a much simpler optical configuration than other devices. In this work, a miniaturized single-beam hybrid optical pumping SERF atomic magnetometer based on a microfabricated atomic vapor cell is demonstrated. Although the optically thin Cs atoms are spin-polarized, the dense Rb atoms determine the experimental results. The enhanced signal strength and narrowed resonance linewidth are experimentally proven, which shows the superiority of the proposed magnetometer scheme. By using a differential detection scheme, we effectively suppress optical noise with an approximate five-fold improvement. Moreover, the cell temperature markedly affects the performance of the magnetometer. We systematically investigate the effects of temperature on the magnetometer parameters. The theoretical basis for these effects is explained in detail. The developed miniaturized magnetometer has an optimal magnetic sensitivity of 20 fT/Hz^1/2^. The presented work provides a foundation for the chip-scale integration of ultrahighly sensitive quantum magnetometers that can be used for forward-looking magnetocardiography (MCG) and magnetoencephalography (MEG) applications.

## Introduction

Among all magnetic sensors, spin-exchange relaxation-free (SERF) atomic magnetometers, which function in high atomic density and low magnetic field environments, have demonstrated the highest magnetic field sensitivity^1–3^. These ultrasensitive magnetometers hold great potential for diverse applications ranging from the detection of dark matter, such as axion-like particles^4^, to biological magnetic field measurement^5–7^. Following the general trend for chip-scale integration, the drawbacks of the orthogonal pump‒probe scheme are gradually emerging. As a derivative, a single-beam absorption-based SERF atomic magnetometer combined with microelectromechanical system (MEMS) technology, which has a much simpler optical arrangement, was developed^8–10^. MEMS atomic vapor cells were first developed by Kitching’s group via silicon microfabrication and silicon-glass anodic bonding techniques^11^. MEMS cells with different microfabrication processes and different geometries have been also successfully fabricated^12–14^. Next, a highly miniaturized atomic magnetometer was constructed on the basis of the developed MEMS cell^8,15^.

The noteworthy features of miniaturized single-beam magnetometers have prompted many new applications, especially in MCG and MEG. However, the large spin polarization gradient across the sensitive head because of strong optical absorption and excess laser amplitude noise caused by the intrinsic detection strategy are considered to be the primary factors limiting their measurement sensitivity and practical applications^16,17^. As a result, various novel methods have been proposed, including counterpropagating pump beams^18^, repumping atomic media^19,20^ and pump‒probe hybrid optical pumping^21–23^. Although the use of hybrid atomic vapor cells filled with different alkali metal atoms has the advantages of a uniform spin polarization distribution and high sensitivity, the hybrid cells are essentially fabricated via conventional glass-blowing techniques and hybrid optically pumped magnetometers are generally configured with a pump‒probe dual-beam arrangement, leading to a considerably complex optical structure^16,24–26^.

In response to the above problems, a miniaturized single-beam hybrid optically pumped SERF magnetometer (SHM) with a microfabricated MEMS cell with an optical differential detection system that utilizes only a single resonant laser source is proposed. By modeling the spin dynamics of two kinds of vapor atoms, we find that the two different vapor atoms have the same electron spin polarizations and spin evolution behavior; thus, they can be interpreted as being of the same spin species. The superiority of this magnetometer is experimentally proven. Moreover, the properties of this magnetometer are significantly dependent on the cell temperature (i.e., alkali atomic density). However, a comprehensive study of the temperature dependence of SHM methods is lacking. Therefore, we systematically investigate the effects of temperature on three important parameters of the integrated magnetometer (magnetic resonance linewidth, output signal amplitude and bandwidth), and the theoretical basis is explained in detail. This work facilitates the development of highly sensitive multichannel chip-scale atomic magnetometer arrays for applications in biomagnetic source imaging.

## Materials and methods

### Microfabrication of MEMS vapor cells

**Fig. 1:**
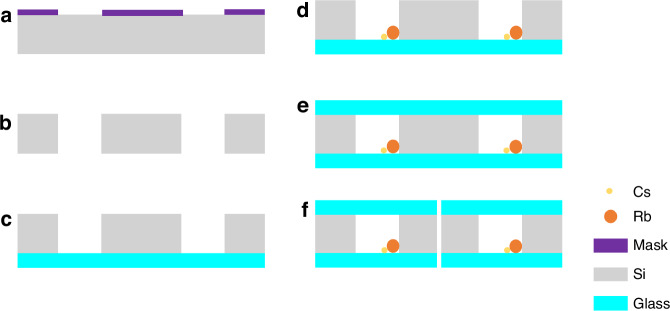
Microfabrication process for the MEMS cells. **a** Patterning of the structure of the cell cavities; **b** etching the Si through-hole cavities via DRIE; **c** first anodic bonding to form the preformed cavities; **d** filling with the hybrid alkali metals and buffer gas; **e** second anodic bonding to complete the MEMS cells; and **f** dicing into single-cell chips

Each MEMS vapor cell is composed of a thick Si wafer sandwiched between two glass wafers. The microfabrication process for MEMS vapor cells begins with a double-sided polished <100>-oriented Si wafer with a thickness of 2 mm and two 500 µm thick borosilicate glass wafers. Figure 1 presents the detailed fabrication procedure for the MEMS cells.

**Fig. 2:**
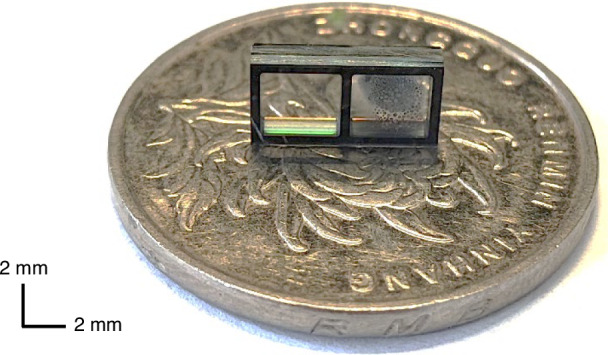
Microfabricated MEMS atomic vapor cell chip

(a) As a mask layer, 500 nm aluminum (Al) is deposited on a cleaned Si wafer via a magnetron sputtering process. Then, the thick AZ4620 photoresist is spin-coated onto the Al layer to pattern an array of cell cavities. Al is chosen as the mask layer because the thick photoresist is insufficient for producing Si through-hole cavities.

(b) Deep reactive ion etching (DRIE) is used to etch through-holes on a 2 mm thick Si wafer. The excellent fabrication precision of the DRIE process guarantees the consistency of the vapor cell structure.

(c) The Si wafer is anodically bonded to the first glass wafer to produce preformed cavities in the atmospheric environment. The anodic bonding parameters include a voltage of 800 V, a temperature of 400 °C and a bonding time of 20 min.

(d) A small droplet of a mixture of Cs and Rb atoms is injected directly into each of the preformed cavities via a controlled pipette in an anaerobic chamber. A controlled buffer gas and quenching gas also fill the cavities. Ne gas is used as the buffer gas to slow the movement of hot alkali atoms toward the cell walls, and N_2_ is used as the quenching gas. The smaller theoretical spin-destruction cross section also gives the magnetometer great potential for higher magnetic field sensitivity.

(e) A second anodic bonding process is implemented to seal the cell cavities and then form the complete array of MEMS vapor cells. To prevent the oxidization of alkali metal atoms during the second anodic bonding and enable better control of the number of atoms, plasma-activated low-temperature anodic bonding is used to seal the MEMS cell. The entire manufacturing process is carried out in an anaerobic glove box.

(f) Finally, the wafer-scale MEMS cells are diced into single-cell chips for subsequent experiments. A fabricated microcell with external dimensions of 8 × 4 × 3 mm^3^ is shown in Fig. 2.

**Fig. 3:**
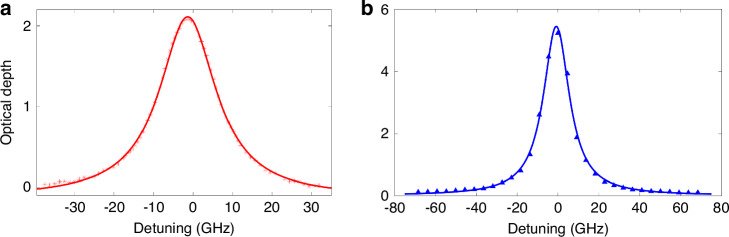
Optical absorption spectra of the hybrid MEMS vapor cell. **a** Cs vapor atom absorption spectrum corresponding to a full width at half maximum (FWHM) of 16.8 GHz; **b** Rb vapor atom absorption spectrum with an FWHW of 17 GHz

We then measure the atomic absorption spectrum to substantiate the existence of the atomic vapors and buffer gas, from which we can determine the density ratio and the pressure of the buffer gas filled into the MEMS cells. Figure 3a, b present the absorption spectra of the Cs and Rb atoms (D1 transition), respectively. The Lorentzian function is used to fit both sets of data. The calculated Ne pressure is approximately 2 amg, and the density ratio of Cs to Rb atoms is approximately 1:2.6 from the fitting results.

### Experimental setup

**Fig. 4:**
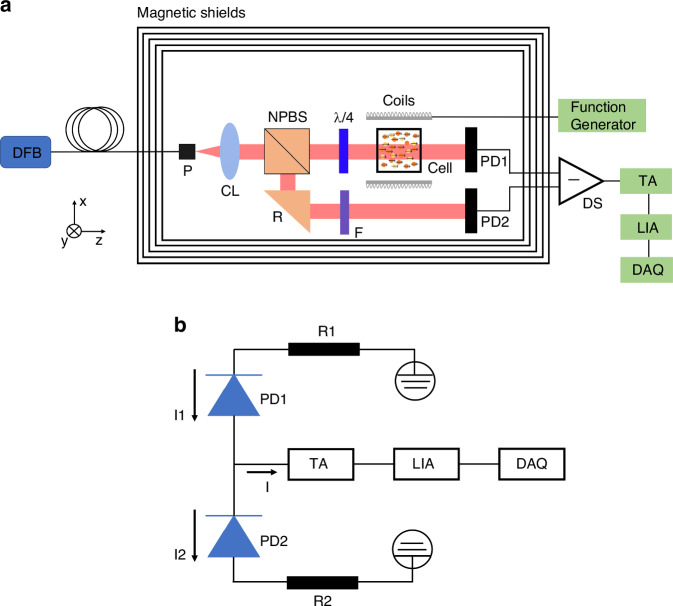
Schematic diagram for the hybrid optical pumping magnetometer. **a** Experimental setup. DFB: distributed feedback laser, P: fiber port, CL: collimating lens, NPBS: nonpolarizing beam splitter, λ/4: quarter waveplate, PD: photodiode, R: reflector, F: variable filter, DS: differential detection system, TA: transimpedance amplifier, LIA: lock-in amplifier, DAQ: data acquisition card. **b** Differential detection system. R: resistance

Schematic diagrams of the experimental setup and the differential detection system for the SHM are shown in Fig. 4. In Fig. 4a, a hybrid MEMS cell, which consists of five layers of μ-metal material shields and an additional ferrite shield layer, is positioned in the central part of the cylinder shields. Tri-axis coils driven by function generators (model: 33500B, Keysight) are available to generate the intended magnetic field. The microfabricated hybrid vapor cell is heated to a specific operating temperature by an additional 1550 nm laser (not shown), and the divergent heating beam, which is optically absorbed by a black filter, ensures a homogeneous cell temperature. The laser heating method completely avoids the effects of stray fields and allows for continuous measurements.

For the optical configuration, a commercially available distributed feedback (DFB) laser (model: DFB pro, Toptica) on-resonance with the D1 transition (894.6 nm) of the Cs atom is transmitted to the sensitive head via a single-mode polarization maintaining fiber. A parallel beam with a diameter of 1.5 mm is obtained from a collimating lens (CL). Then, the collimated light is separated by a nonpolarizing beam splitter (NPBS) into two parts: a measurement beam and a reference beam. The measurement beam is converted into circularly polarized light by a quarter waveplate (λ/4) and is used to optically pump thin Cs atoms through the process of photon angular momentum transfer. Afterward, thick Rb atoms are also spin-polarized by fast collisions with the polarized Cs atoms, a process that can lead to more uniform spin polarization of the thick Rb atoms throughout the whole sensor head. Two identical silicon photodiodes (PD1 and PD2) are exploited to receive the two beams, following which the custom-made differential detection system (DS) works by subtracting the light between the functionalized light beam and reference light beam (i.e., the top beam and bottom beam), as shown in Fig. 4b. Finally, the differential signals pass in turn through a transimpedance amplifier (TA, model: PDA200C, Thorlabs), which converts the photocurrent to voltage; a lock-in amplifier (LIA, model: MFL1, Zurich Instruments), which extracts the first harmonic component of the modulated signal; and a data acquisition card (DAQ, model: PCI4461, National Instruments) for experimental data processing.

The process of differential detection is described in detail as follows. First, the light entering PD2 is blocked. Then, the light power of the measurement beam is adjusted so that the electron spin polarization reaches 50%. The photocurrent I1 passing through the hybrid cell is measured by PD1, which is the output signal of the magnetometer system, including the DC background offset and noise of approximately 90 V/Hz^1/2^. Finally, the light irradiating PD2 is turned on, and the total photocurrent I (i.e., I = I1-I2) may be significantly attenuated by keeping the direction of the variable filter (F) well controlled; thus, cancellation of the detected background offset is achieved while the optical power noise is greatly suppressed.

## Results

### Theoretical feasibility

Alkali metal atomic magnetometers typically operate with spin-polarized electrons that undergo Larmor precession around the imposed magnetic field to obtain external field information. For the hybrid optical pumping scheme, the second optically dense atoms *A* are polarized by the other thin atoms *B* pumped by a resonant laser source through a fast spin-exchange collision process. The general evolution behavior of the atomic spin at a low magnetic field and high atomic density can be well represented by a group of Bloch equations^1,22^:

1 $$\left[\begin{array}{c}{{\cdot }\atop{\displaystyle{P}_{B-x}}}\\ {{\cdot }\atop{\displaystyle{P}_{B-y}}}\\ {{\cdot }\atop{\displaystyle{P}_{B-z}}}\end{array}\right]=\frac{1}{{Q}_{B}(P)}\left({g}_{s}{\mu }_{B}\left[\begin{array}{c}{P}_{B-x}\\ {P}_{B-y}\\ {P}_{B-z}\end{array}\right]\times \left[\begin{array}{c}{B}_{x}\\ {B}_{y}\\ {B}_{z}\end{array}\right]+{R}_{op}\left[\begin{array}{c}-{P}_{B-x}\\ -{P}_{B-y}\\ 1-{P}_{B-z}\end{array}\right]+{R}_{ex}^{A-B}\left[\begin{array}{c}{P}_{A-x}\\ {P}_{A-y}\\ {P}_{A-z}\end{array}\right]-\left[\begin{array}{c}\frac{{P}_{B-x}}{{T}_{B-2}}\\ \frac{{P}_{B-y}}{{T}_{B-2}}\\ \frac{{P}_{B-z}}{{T}_{B-1}}\end{array}\right]\right)$$

2 $$\left[\begin{array}{c}{{\cdot }\atop{\displaystyle{P}_{A-x}}}\\ {{\cdot }\atop{\displaystyle{P}_{A-y}}}\\ {{\cdot }\atop{\displaystyle{P}_{A-z}}}\end{array}\right]=\frac{1}{{Q}_{A}(P)}\left({g}_{s}{\mu }_{B}\left[\begin{array}{c}{P}_{A-x}\\ {P}_{A-y}\\ {P}_{A-z}\end{array}\right]\times \left[\begin{array}{c}{B}_{x}\\ {B}_{y}\\ {B}_{z}\end{array}\right]+{R}_{ex}^{B-A}\left[\begin{array}{c}{P}_{B-x}\\ {P}_{B-y}\\ {P}_{B-z}\end{array}\right]-\left[\begin{array}{c}\frac{{P}_{A-x}}{{T}_{A-2}}\\ \frac{{P}_{A-y}}{{T}_{A-2}}\\ \frac{{P}_{A-z}}{{T}_{A-1}}\end{array}\right]\right)$$

where the subscripts *A* and *B* represent different kinds of vapor atoms; $P(\cdot )$_*x,y,z*_ represent the evolution of the electron spin polarization over time in three perpendicular directions; *Q*(*P*) represents the nuclear slowing-down factor; *g*_*s*_ represents the *g* factor; *μ*_*B*_ represents the Bohr magneton; *B*_*x,y,z*_ represent the tri-axial magnetic field; *R*_*op*_ represents the optical pumping rate; *R*_*ex*_ represents the spin-exchange collision rate; and *T*_1,2_ represent the longitudinal relaxation time and transverse relaxation time, respectively.

The equilibrium spin polarization of the Bloch equations for B and A vapor atoms can be given by

3 $${P}_{0}^{B}=\frac{({R}_{relA}+{R}_{ex}^{B-A}){R}_{op}}{({R}_{relB}+{R}_{op}+{R}_{ex}^{A-B})({R}_{relA}+{R}_{ex}^{B-A})-{R}_{ex}^{B-A}{R}_{ex}^{A-B}}$$

4 $${P}_{0}^{A}=\frac{{R}_{ex}^{B-A}{R}_{op}}{({R}_{relB}+{R}_{op}+{R}_{ex}^{A-B})({R}_{relA}+{R}_{ex}^{B-A})-{R}_{ex}^{B-A}{R}_{ex}^{A-B}}$$

where *R*_*rel*_ is the relaxation rate. Under SERF regime conditions, the spin-exchange collision rate ${R}_{ex}^{{B}-{A}}$ and the spin relaxation rate *R*_*relA*_ are ~10^5^ s^−^^1^ and ~10^2 ^s^−^^1^, respectively; that is, the spin-exchange rate is much greater than the A atomic relaxation rate $({R}_{ex}^{{B}{-}{A}}\,>>\,{R}_{relA})$. Therefore, an astonishing conclusion that the spin polarizations of the two alkali atoms are approximately the same (*P*_*A*_ ≈ *P*_*B*_) can be reliably drawn, which indicates that the two different vapor atoms have the same spin evolution behavior. Eventually, they can be treated as the same spin species^27,28^. This behavior is mainly attributed to the very fast spin-exchange collisions between *A* and *B* atoms. As a result, even though the optically thin atoms *A* are optically pumped by a resonant laser source, the dense atoms *B* polarized by fast spin-exchange collisions have a dominant effect on the experimental results. Consequently, several attractive experimental features, including higher signal strength related to high atomic density, narrower linewidth dominated by light but dense atoms, more uniform spin polarization due to the optical pumping of thin atoms and a much simpler optical configuration owing to the merging of pumping and probing beams, can be achieved in this demonstrated magnetometer.

In the single-beam experimental arrangement, where the pumping and probing beams are merged into a single beam, optical absorption is detected to characterize the magnetic field. When the pumping light propagates through the hybrid MEMS cell along the *z*-direction, the attenuation of the optical pumping rate is given by^29,30^

5 $$\frac{d{R}_{op}}{dz}=-{n}_{B}\sigma (\nu ){R}_{op}(1-{P}_{B-z})$$

where *I*_*B*_ is the light power, *n*_*B*_ is the *B* atomic density and *σ*(*v*) is the photon absorption cross section. The solution of Eq. (5) can be obtained by considering two cases^31,32^:

6.1 $${R}_{op}={K}_{1}{I}_{B}{e}^{-{n}_{B}\sigma (\nu )L(1-{P}_{B-z})}\,{\rm{for}}\,T\le C$$

6.2 $${R}_{op}={K}_{2}\frac{{R}_{rel}}{\sigma (\nu )}W\left[\frac{\sigma (\nu ){I}_{0}}{{R}_{rel}}\exp \left(\frac{\sigma (\nu ){I}_{0}}{{R}_{rel}}-{n}_{B}\sigma (\nu )z\right)\right]\,{\rm{for}}\,T > C$$

where *K*_*1*_ and *K*_*2*_ are proportional parameters, *L* is the length of the light‒atom interaction, *W(z)* is the Lambert W-function, *I*_0_ is the incident power, *R*_*rel*_ is the spin depolarization rate and *T* is the cell temperature. When the *B* atomic density is small, the amount of light pumped through the cell can be considered constant. However, strong optical absorption arises under high-atomic-density conditions. The demarcation point *C* is also determined here. The total magnetic resonance linewidth Δ*B* can be expressed as

7 $$\Delta B=\frac{{R}_{op}+{R}_{sd}+{R}_{wall}+{R}_{\mathrm{mod}}+{R}_{se}}{{\gamma }^{e}}$$

where *γ*^*e*^ is the gyromagnetic ratio of the electron, *R*_*sd*_ is the spin-destruction relaxation rate, *R*_*wall*_ is the cell wall collision rate, *R*_*mod*_ is the spin relaxation rate due to the applied modulation field *B*_*x*_ = *B*_*x0*_ + *B*_*m*_ *cosωt* and *R*_*se*_ is the spin-exchange relaxation rate, which can be ignored when the vapor atoms are in the SERF regime.

**Fig. 5:**
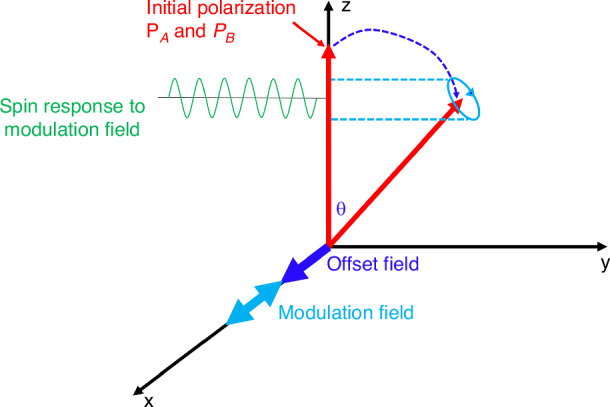
Schematic diagram of the single-beam magnetometer response to a magnetic field

Furthermore, for a single-beam atomic magnetometer, there will be a large background offset in the detected signal caused by the intrinsic detection scheme of optical absorption. The applied modulation magnetic field also results in a sinusoidal signal being superimposed on the DC offset signal, as shown in Fig. 5.

When the modulation magnetic field is considered, the change in the output light power caused by the modulation field is

8 $$\delta I={K}_{3}\frac{{I}_{H}}{2{R}_{tot}}{\gamma }^{e}{n}_{B}\sigma (\nu )L{B}_{x0}{e}^{-0.5{n}_{B}\sigma (\nu )L}+\delta {I}_{DC}+\delta N$$

where *K*_*3*_ is a coefficient related to the modulation parameters, *I*_*H*_ is the light power required at 50% spin polarization, *δI*_*DC*_ is the DC background offset, and *δN* is the optical noise due to the laser amplitude fluctuation and common mode noise. The last two terms can be effectively suppressed by means of a differential detection system, which is accomplished by subtracting the measurement optical path from a reference path with an almost identical optical component. As analyzed, the hybrid optical pumping magnetometer requires higher pumping power because of spin polarization transportation. Therefore, to maintain the optimal polarization of 50%^9,10,24^, a higher optical power than that of a single-species atomic magnetometer is necessary, which undoubtedly leads to greater optical noise. In addition to the choice of ultrastable resonant laser source and optical components, the developed active differential detection system offers very fascinating advantages in hybrid optical pumping magnetometers.

### Superiority of the SHM

**Fig. 6:**
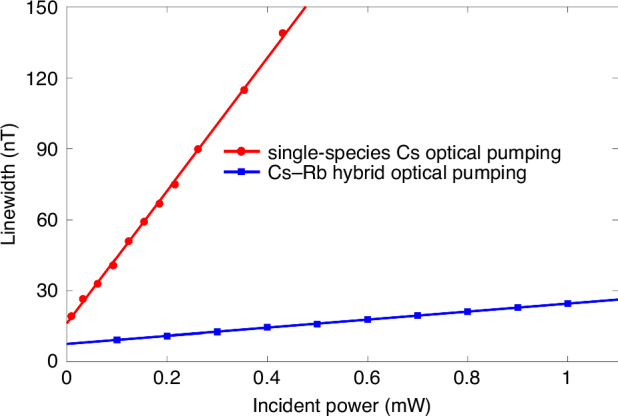
Linear relationship between the incident power and magnetic resonance linewidth

**Fig. 7:**
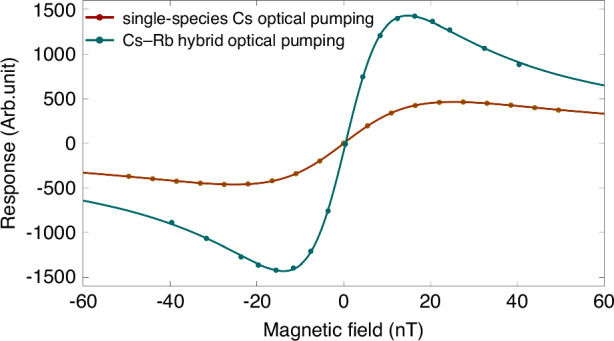
Zero-field resonance effects of a single-species Cs atomic magnetometer and the miniaturized Cs-Rb SHM

We first verify that two different vapor atoms have the same spin evolution behavior and can be regarded as being of the same spin species via a single-species Cs magnetometer and the miniaturized Cs-Rb SHM. To ensure the reliability of the experiments, the microfabrication processes of the single-species Cs atomic cell and the Cs-Rb hybrid cell are identical. Under the same experimental conditions, i.e., the same cell size, a Cs atomic density of 3.1 × 10^13 ^cm^−^^3^, and a modulation field with a modulation frequency of 990 Hz and modulation amplitude of 180 nT applied in the *x*-direction, the relationships between the incident light power and zero-field resonance linewidth are measured and fitted linearly, as shown in Fig. 6, from which the corresponding linewidths are 16.2 nT and 7.6 nT by extrapolation to the zero-power point in the two experimental configurations. Then, the zero-field resonance curves are acquired by sweeping the transverse magnetic field crossing over zero and recording the output signal when the spin polarization is 50%. The experimental results are presented in Fig. 7, from which it can be clearly observed that the desired experimental results are obtained via the SHM, including higher signal amplitudes, larger scale factors and narrower magnetic resonance linewidths. These are necessary to achieve high magnetic field sensitivity for the SHM. The theoretical basis behind these experimental results is that although fewer Cs atoms are optically pumped by a DFB laser, the optically dense Rb atoms play a dominant role in the experiment because they have the same spin evolution; that is, we can counterintuitively think that the high-density Rb atoms are actually probed. Therefore, the higher signal strength contributes to the high Rb atomic density, and the narrower magnetic linewidth can account for the smaller spin-destruction cross section.

However, higher pumping power is also required in the hybrid optical pumping magnetometer, which undoubtedly introduces an excessive amount of laser amplitude noise; this noise is treated as the primary source of noise in the SHM. Moreover, the single-beam magnetometer uses optical absorption to characterize the magnetic field, and there is a large background offset in the detected signal. Therefore, a differential detection scheme is utilized. The measurement beam through the MEMS hybrid vapor cell and a reference beam with approximately the same optics as the measurement light path are subtracted, which achieves approximately five times the laser noise suppression effect.

**Fig. 8:**
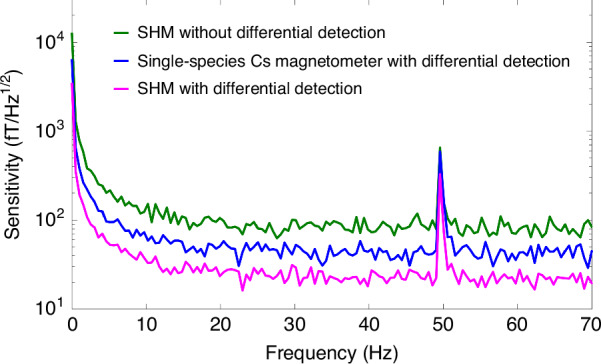
Noise spectral densities of the magnetometers with different configurations. The sensitivity is 42 fT/Hz^1/2^ for the single-species Cs magnetometer, whereas the sensitivity is 20 fT/Hz^1/2^ for the miniaturized SHM

Then, the magnetic field sensitivity is calibrated. The output out-of-phase component of the lock-in amplifier (LIA) signal is recorded for a 20 s sampling time at a sampling rate of 3.348 kSa/s. The power spectral density of the output signal is directly calculated and averaged for 1-Hz bins. We then divide it by the frequency response to normalize the magnetic noise spectrum, in which case the noise floor can directly indicate the magnetic sensitivity, as shown in Fig. 8. Eventually, an ultrahigh magnetic field sensitivity of 20 fT/Hz^1/2^ in the range of 15–70 Hz is achieved via our SHM. The sensitivity peak at 50 Hz in Fig. 8 is caused by industrial frequency interference.

**Fig. 9:**
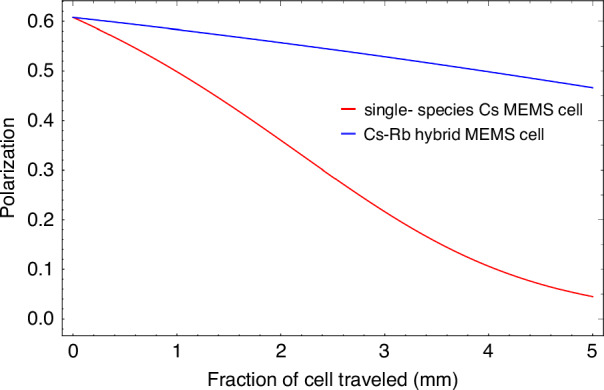
Spin polarization distributions through the Cs-Rb hybrid MEMS cell and single-species Cs MEMS cell. The total atomic density of 3.1 × 10^13 ^cm^−^^3^ and the pumping rate (R_OP_ = 1.5R_rel_) at the front of the cell are held constant

To illustrate the relatively homogeneous spin polarization distribution, a theoretical comparison is performed between the single-species Cs magnetometer and the proposed SHM. The simulation results are shown in Fig. 9, from which it can be clearly seen that the distribution of the spin polarization is much more uniform.

### Temperature dependence

**Fig. 10:**
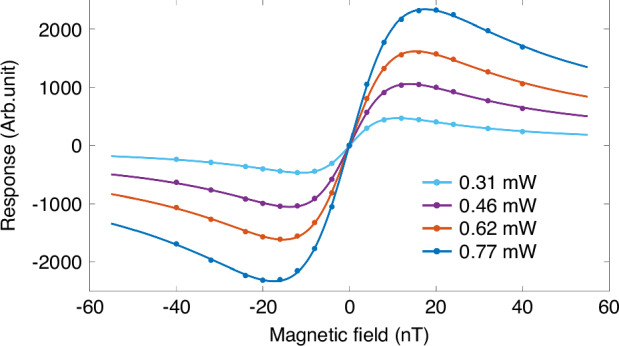
Magnetic resonance response under different incident powers

**Fig. 11:**
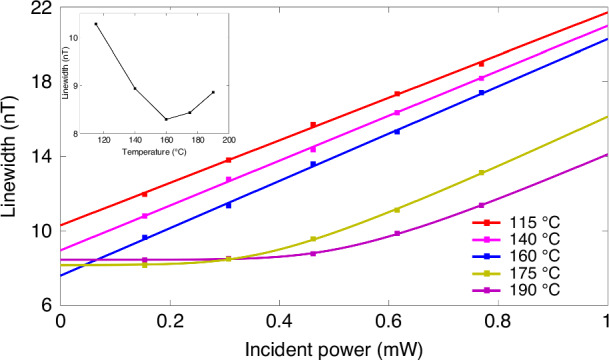
Zero-field resonance linewidth as a function of the incident power at different temperatures. The inset is the linewidth corresponding to the extrapolation to zero power

The comprehensive influence of the cell temperature on the magnetometer parameters is investigated. The demodulated response of atomic spins to the transverse magnetic field is first acquired at a fixed temperature of 160 °C, as presented in Fig. 10. These experimental data are fitted by the dispersive function:

9 $$S({B}_{x})={k}_{0}\frac{{B}_{x}-{B}_{x0}}{\Delta {B}^{2}+{({B}_{x}-{B}_{x0})}^{2}}$$

where *k*_0_ is the scale factor and *B*_*x*0_ is the residual field in the *x*-direction. With increasing optical power, the resonance linewidth gradually broadens, which is the result of optical pumping. Figure 10 shows the relationship between the resonance linewidth and incident light power, which is measured at different cell temperatures. The resonance linewidth is proportional to the incident power when the temperature is low, as shown by the red and magenta curves in Fig. 11. This relationship gradually deviates from the linear model as the temperature increases. In particular, when the cell temperature is approximately 160 °C, the same R-squared can be obtained by overlapping the experimental data via Eqs. (6.1) and (6.2), and this temperature can also be considered the demarcation point *C*. The linewidth follows the Lambert W-function of the incident power when the temperature is greater than 160 °C, and the data overlap with the Lambert W-function, with a fitted R-squared better than 99.95%. This phenomenon is induced by two main factors. The first factor is that strong optical absorption occurs as the cell temperature increases. The second is that the spin-exchange rate between the Rb and Cs atoms rapidly increases at elevated temperatures, resulting in the condition that two nominally distinct spin species with the same spin polarization behavior can be regarded as one spin species being satisfied more easily. As a result, the optically thick Rb atoms have a primary function in the experiment. Moreover, the extrapolation of the resonance linewidth to zero power indicates a minimum relaxation rate except for the optical pumping rate in the inset of Fig. 10, from which we can obtain an optimal operating temperature of 160 °C, which corresponds to a total spin relaxation rate of 1340 s^−^^1^.

**Fig. 12:**
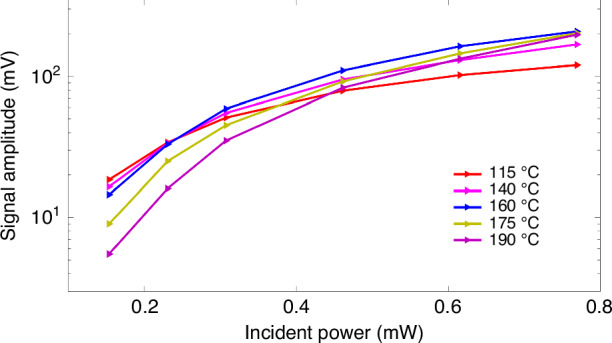
Signal amplitude under different temperatures

The output signals as a function of the incident optical power are also measured, as shown in Fig. 12. The detailed measurement process is as follows. A calibration field with an oscillating intensity of 0.8 nTrms and a frequency of 30 Hz is applied to the SHM in the direction perpendicular to the laser beam; then, the output signal amplitude of the out-of-phase component of the LIA is recorded simultaneously. A general rule is that with increasing incident light power, the output signal increases gradually. However, there are two distinguishing features: i. Under very low optical power, the output signal of the magnetometer is larger at lower temperatures than at higher temperatures. ii. Excessive temperatures also lead to a reduction in the output signal amplitude. These experimental results may be explained by the atomic density and the transfer process of spin polarization. As demonstrated in the theory section, the SHM demands a higher incident power. The spin polarization rate, which increases almost linearly with the incident power, is the main reason for the high signal amplitude, despite the lower atomic density at the lower temperature of 115 °C. Nevertheless, strong absorption is not sufficient to compensate for the slow increase in spin polarization, leading to a reduction in the output signal at very high temperatures. Moreover, there are good reasons to believe that a further increase or decrease in temperature will cause a decrease in the output signal amplitude.

**Fig. 13:**
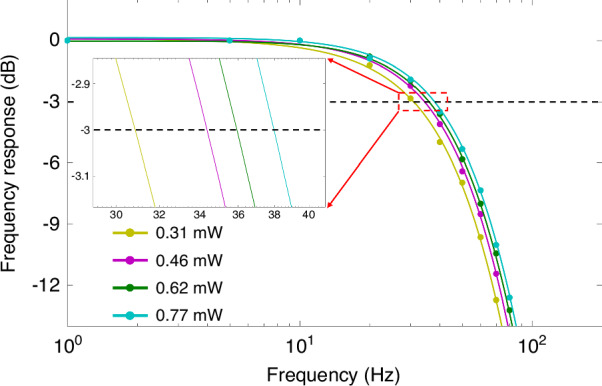
Normalized frequency response curves under different incident optical powers at 160 °C

**Fig. 14:**
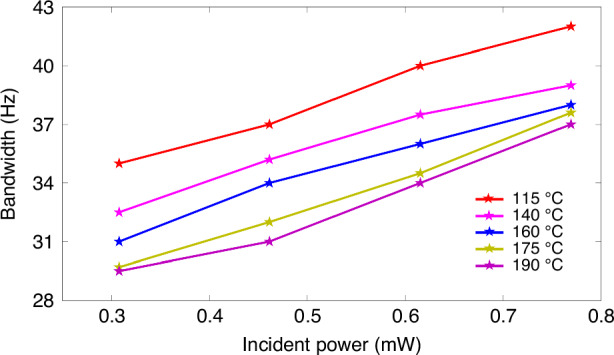
Bandwidth as a function of incident power at different temperatures

Finally, the normalized frequency response, which behaves as a first-order low-pass filter, *R*(*f*)*=K*/(*f*^*2*^_*s*_ + *f*^*2*^_*3* *dB*_)^1/2^, where *K* is a constant, *f*_*s*_ is the frequency of the applied magnetic field, and *f*_*3dB*_ is the magnetometer bandwidth, is obtained under different incident optical powers at 160 °C, as shown in Fig. 13. The bandwidth of the magnetometer, i.e., the frequency point at a gain of −3 dB, can be easily determined. The bandwidth is proportional to the magnetic resonance linewidth or total spin relaxation rate. Therefore, the bandwidth gradually expands under different incident powers from 0.31 to 0.77 mW. Figure 14 shows the relationship between the incident power and the bandwidth at different temperatures. The increased bandwidth at low temperatures can be attributed to the high spin relaxation rate. Specifically, the SERF regime may be invalid at low atomic densities, introducing an additional spin-exchange relaxation rate. As the temperature increases, the atoms gradually enter the SERF state, leading to a lower bandwidth.

## Discussion

The magnetic sensitivity δ*B* of the atomic magnetometer depends on the signal-to-noise ratio (SNR) as well as the magnetic resonance linewidth Δ*B* (i.e., δ*B*∝Δ*B/*SNR). The SNR improvement is guaranteed by the optical differential detection system and the same spin evolution behavior of optically thick Rb and thin Cs atoms. The resonance linewidth, which is proportional to the total spin relaxation rate, is also one of the most important parameters for determining the magnetometer measurement sensitivity. The heavier Cs atoms, as optically thin atoms, are pumped by a resonant laser resource, whereas the lighter, dense Rb atoms dominate the experimental results in the hybrid MEMS cell, which leads to a narrower magnetic resonance linewidth because of the smaller spin-destruction collision cross sections. Another advantage is that the uniform spin polarization throughout the sensitive head is also scored because of the optically thin atoms being pumped. Owing to these attractive advantages, the proposed magnetometer finds its niche in the field of atomic magnetometry, especially for biomagnetic field imaging applications, such as MCG and MEG. Moreover, the cell temperature is one of the two most important conditions in hybrid optically pumped SERF atomic magnetometers. The cell temperature not only affects the output signal strength but also directly determines the dynamic spin evolution behavior of two different alkali metal atoms. The investigation of the temperature dependence of the magnetometer enables a better understanding of the spin evolution processes of the two atoms and the optimization of the magnetometer operating temperature.

## Conclusions

We developed a miniaturized single-beam integrated hybrid optical pumping SERF atomic magnetometer with a MEMS hybrid cell accompanied by an optical differential detection system. We experimentally verified the fascinating features of this magnetometer, including a high signal amplitude, narrow magnetic resonance linewidth and simple optical arrangement. Furthermore, the comprehensive effect of the cell temperature on the magnetometer performance was studied, which can help guide further enhancements in the sensitivity of chip-scale atomic magnetometers. In the future, we intend to construct a multichannel magnetometer array for MCG and MEG on the basis of the demonstrated miniature hybrid optical pumping atomic magnetometer.

## Data Availability

The data underlying the results presented in this paper are not publicly available at this time but may be obtained from the authors upon reasonable request.
